# Rising to the challenge: lessons learnt from the Global Women’s Research Society (GLOW) conference for women’s and newborn health in the context of global crises

**DOI:** 10.1080/26410397.2025.2525656

**Published:** 2025-06-30

**Authors:** Clara Calvert, Sagarika Kaushal, Aduragbemi Banke-Thomas, Zeina Jamaluddine, Brian Matovu, Jennifer Riches, Robert Ssekitoleko, Wendy Graham, Rosemary Townsend

**Affiliations:** a Senior Lecturer, Usher Institute, University of Edinburgh, Edinburgh, UK.; b Medical student, Edinburgh Medical School, University of Edinburgh, Edinburgh, UK; c Associate Professor, Faculty of Epidemiology and Population Health, London School of Hygiene and Tropical Medicine, London, UK; d Assistant Professor, Faculty of Epidemiology and Population Health, London School of Hygiene and Tropical Medicine, London, UK; e Biomedical Engineer, Center for Design, Innovation and Translational Excellence, Biomedical Engineering Unit, School of Biomedical Sciences, Makerere University, Makerere, Uganda; f Clinical Research Fellow, Maternal and Fetal Health Group, Malawi-Liverpool-Wellcome Research Programme, Blantyre, Malawi; Department of Women's and Children's Health, University of Liverpool, Liverpool, UK; h Professor, Faculty of Epidemiology and Population Health, London School of Hygiene and Tropical Medicine, London, UK; i Senior Clinical Lecturer, Centre for Reproductive Health, University of Edinburgh, Edinburgh, UK

Over the past 25 years, there has been remarkable progress in reducing maternal, stillbirth and neonatal mortality.^1–3^ However, emerging data suggest that this progress is stalling and, in some regions, even reversing.^1,4^ This worrying trend is being driven by some of the major global crises. These include climate breakdown, conflict, antimicrobial resistance and deteriorating mental health, all of which often intersect to affect the same particularly vulnerable populations. There is a wealth of evidence linking these crises to adverse outcomes in pregnant people and their babies^5–8^ and, consequently, a need to consider how, as a community of clinicians, researchers, advocates and creative artists working in reproductive health, we can try to mitigate the impacts of these global crises to ensure that all women and their babies survive and thrive. This consideration was at the core of what we set out to achieve at the most recent conference for the Global Women’s Research Society (GLOW), a society established over 10 years ago to facilitate education and networking among researchers and clinicians working on reproductive, maternal and newborn health globally. The ninth GLOW conference, hosted by the University of Edinburgh in collaboration with Napier University in September 2024, focussed on the effects of the ongoing global crises of climate change, infectious diseases, mental health, and conflict and migration on women’s, reproductive and newborn health. To ensure that the conference was accessible to people from across the globe, the conference was delivered as hybrid, with both online and in-person attendance options. Additionally, targeted travel and conference participation funding support for conference participants from low- or middle-income countries (LMICs) was instituted. In total, 265 people registered to attend the conference of which half were from a LMIC. The complete conference programme is available at http://www.glowconference.org/conference-programme.html and all the conference materials are available to view at https://app.medall.org/event-listings/glow-2024-rising-to-the-challenge-women-and-newborn-health-in-the-context-of-global-crises.

The evidence presented at the GLOW conference on the impacts of global crises on reproductive, maternal and newborn health was sobering. Still, it was clear the community is dedicated to championing change, with countless examples over the 2-day conference of successful innovation and partnerships that are “rising to the challenge” to meet these crises head-on. This commentary aims to showcase some of the examples of successful innovation and partnership from the GLOW community and consider how we can harness the lessons to help tackle global health crises from our perspective as a group of members from the GLOW 2024 organising committee or presenters at the conference. We will focus on three of the recurring themes that emerged at the GLOW conference, specifically: (1) the power of routine data in crisis situations, (2) the potential for new technologies and (3) the importance of partnerships across different disciplines, between different professions and with those most affected by these crises to ensure any solutions are acceptable and practicable.

In crisis situations, such as war, disease outbreaks or climate disasters, data is rarely at the forefront of anyone’s mind. However, throughout the GLOW conference, it was made clear how **routinely available data** can be a powerful tool to rapidly quantify the impacts of crises on maternal, newborn and reproductive health. This evidence, in turn, can be used to inform the disaster response, understand vulnerabilities in the health system, and advocate for resources to try and avert or mitigate the impact on women and their babies. We saw the devastating impacts of the war in Gaza on pregnant people and their babies through quantified pre-war data and projection models for differing scenarios in the war.^9^ For example, under the escalation scenario, which modelled a decrease in access to hospitals among other interventions, there was a projected increase in the maternal mortality ratio from 23 maternal deaths per 100,000 live births pre-war to 103 deaths per 100,000 live births since the war started – and these estimates do not include any deaths projected to have occurred due to traumatic injuries among pregnant and postpartum people. These models projected that should the conflict escalate, as it has, maternal mortality (excluding traumatic injuries) would revert to a level last seen in Gaza in the 1990s. The data from Gaza have been used as an advocacy tool,^10–12^ raising awareness of the impacts of the conflict on the whole population. Similarly, another presentation at the conference used routine aggregated data available from a national surveillance platform in Malawi to quantify the catastrophic impacts of Cyclone Freddy in February–March 2023 on maternal healthcare provision.^13^ This work showed an immediate reduction in service utilisation following the cyclone, including, for example, a 34% decline in the number of antenatal care visits in the post-cyclone compared to the pre-cyclone period. Unfortunately, the availability and quality of routine data are not adequate across all settings, and there are many crisis scenarios where it has not been possible to quantify the impacts on the population, including in the ongoing war in Sudan,^14^ where deaths and injuries go uncounted, and the absence of data hinders recovery of health systems. Even in settings with robust data systems, major crises almost inevitably lead to the deterioration of any routine data collection in place and at a time when they are so critically required. However, even with deterioration in data availability and quality, having pre-existing robust data systems is important when crises occur as they may be restored and adapted to facilitate the most urgent data needs. Ensuring robust data infrastructure and, where possible, the near real-time availability of routine data, and the requisite skills to interrogate these, is essential as part of strategies to deal with ongoing and future global crises. However, data alone will not protect people from the impact of crises and these data need to be used to inform action to ease the suffering of those made vulnerable by the crisis.

A second key theme to emerge at the GLOW conference was the potential for **new technologies** to support the delivery of women’s and newborn healthcare in crisis situations. This is contingent on our ability to bring durable and reliable tools to women and babies rather than placing the burden of travelling on them to reach health facilities in challenging circumstances. At GLOW, there were several exciting and promising examples of new technologies being applied in high need situations, taking innovative solutions to where they can deliver the most impact. Some examples include: (1) the SMARThealth Pregnancy program using tablets to support community health workers to provide guideline-based maternal care in India;^15^ (2) the CRADLE Vital Signs Alert device in Sierra Leone to manage hypertension and shock in pregnancy;^16^ (3) wireless physiological monitoring tools designed to reduce workload and increase patient safety in low resource settings;^17^ and (4) a urine-based point-of-care diagnostic strip for pre-eclampsia that is envisioned to empower women to take charge of their health in low- and middle-income countries.^18^ It was clear, however, that there remain challenges in the sustainable and equitable implementation of new technologies, including ensuring affordability and, consequently, access by the most vulnerable in the population, and the long development pipeline leading to delays in wide-scale implementation.^19^ The analogy of mobile technology “leapfrogging” landline-based telecommunications in low-income settings is only partially applicable to healthcare innovation where equity of access is critical for public benefit. As such, new technologies can only form part of the solution and must be embedded within essential rights-based strategies for sustainable development that seek to ensure all women and their babies survive and thrive physically, mentally, socially and economically. The development of successful technologies requires that clinicians, researchers and the public must work with engineers, innovators and regulators to develop contextually relevant and effectively translated devices, as exemplified in the Centre of Design, Innovation and Translational Excellence (CITE) at Makerere University.^20^

The third key theme emerging from the GLOW conference and a consistent message across all sessions in the conference was how essential **partnerships** are as we work together to mitigate the impact of crises on women’s and newborn health. The CITE presentation was an excellent example of a successful, ongoing, interdisciplinary, international partnership. Working in silos is not likely to bring around the required solutions, actions or buy-in, and we must rely on genuinely collaborative partnerships. There were many inspiring examples of such collaboration, with different communities coming together to bring about meaningful change: interdisciplinary partnerships, international partnerships, private and public sector partnerships, partnerships bringing together science and arts, and partnerships ensuring meaningful inclusion of the community and/or patient voice. We saw how academics working in collaboration with Google were able to use internal Google data to measure travel time to emergency obstetric care in urban areas of Nigeria, considering factors like real-life traffic differentials. This partnership led to much closer-to-reality estimates of travel time for pregnant people in urban Nigeria and, to ensure the utility of the work in planning services, has been made publicly available as a Google digital dashboard.^21^ We should also highlight the work of the Lifeline Nehemiah Projects,^22^ a grassroots organisation based in Freetown, Sierra Leone, with a strong focus on community engagement. They have established a mentoring maternal group to support pregnant adolescents, and the results suggest that this has not only had an impact on the adolescents but has had a wider positive impact on the community’s mindset.^23^ We also were presented with innovative approaches to engagement, developed through partnerships between researchers and clinicians, with creative artists and performers, including the co-creation of an interactive walk exploring the legacy of birth and women’s health in the host city, Edinburgh, for this year’s GLOW conference.^24^ All the examples of the partnerships presented at GLOW were reassuring, demonstrating how we are learning to work together as a diverse community, and this can lead to creative solutions – but there is always space to do better. In particular, we believe in an increasing role for South-South collaborations and, as illustrated by the study in Sierra Leone,^23^ it is essential that all researchers work closely with affected communities to hear what these communities need to facilitate responsive rather than directive research design.

As we conclude this commentary, it should be highlighted that many of the crises we are facing were not and are not inevitable. Nevertheless, when these crises arise, the global community must advocate for the rights of women and babies, as particularly vulnerable groups, drawing on a diverse set of skills including those of clinicians, epidemiologists, data scientists, social scientists, and health systems experts. Major crises have pervasive and complex impacts on populations and advocacy must be based on high-quality evidence drawn from across different fields and must be done in collaboration with affected communities, ensuring that their voices, experiences, and needs are at the forefront of any action. In this commentary, we have highlighted the importance of having strong data systems in place globally to gauge the impacts of crises on health and ensure that resources reach those who need them the most, the importance of developing new technologies that are durable and reliable in crisis situations, and the necessity of working in partnerships. A recurring thread throughout the GLOW conference was the disproportionate burden of crises on marginalised women and their babies. This was an important reminder that it is our responsibility as part of the GLOW community to ensure that we do not leave these vulnerable women behind and that we always carefully think about who may be missing from routine data, who might not be able to access technologies and who might not be reached as part of stakeholder/community engagement – and work out how to overcome these barriers. We have shown here just some of the ways in which our community has stepped up – using routine data, developing new technologies and working in partnerships – and we left the GLOW conference with some optimism that we can contribute to efforts to save women’s and babies lives, even under the most challenging of circumstances, and will continue to work together to identify new ways to address global crises. The next GLOW conference will be in London in 2026 and this will provide an excellent opportunity to bring together actors working on reproductive, maternal and newborn health from across the globe again and critically review the GLOW community’s continuing contribution to addressing the prevailing and emerging challenges.

## Provenance statement

This article was not commissioned and went through internal review.
